# Wearable sensor derived decompensation index for continuous remote monitoring of COVID-19 diagnosed patients

**DOI:** 10.1038/s41746-021-00527-z

**Published:** 2021-11-08

**Authors:** Dylan M. Richards, MacKenzie J. Tweardy, Steven R. Steinhubl, David W. Chestek, Terry L. Vanden Hoek, Karen A. Larimer, Stephan W. Wegerich

**Affiliations:** 1 physIQ Inc., 200 W Jackson Blvd Suite 550, Chicago, IL USA; 2 University of Illinois Health, Chicago, IL USA

**Keywords:** Machine learning, Computational platforms and environments, Hardware and infrastructure

## Abstract

The COVID-19 pandemic has accelerated the adoption of innovative healthcare methods, including remote patient monitoring. In the setting of limited healthcare resources, outpatient management of individuals newly diagnosed with COVID-19 was commonly implemented, some taking advantage of various personal health technologies, but only rarely using a multi-parameter chest-patch for continuous monitoring. Here we describe the development and validation of a COVID-19 decompensation index (CDI) model based on chest patch-derived continuous sensor data to predict COVID-19 hospitalizations in outpatient-managed COVID-19 positive individuals, achieving an overall AUC of the ROC Curve of 0.84 on 308 event negative participants, and 22 event positive participants, out of an overall study cohort of 400 participants. We retrospectively compare the performance of CDI to standard of care modalities, finding that the machine learning model outperforms the standard of care modalities in terms of both numbers of events identified and with a lower false alarm rate. While only a pilot phase study, the CDI represents a promising application of machine learning within a continuous remote patient monitoring system.

## Introduction

To date, more than 130 million people have been infected with SARS-CoV-2 (the virus that causes COVID-19)^1^. Of those infected, approximately one in five are at risk for severe decompensation, resulting in hospitalization^2–4^. While much has been learned over the past year about the transmission of the virus, little is known about the factors that predict a symptom-free disease course versus an acute disease course requiring interventions like oxygen, hospitalization and/or mechanical ventilation. Determining which patient will decompensate (have worsening COVID-19 illness) in order to appropriately triage to home or hospital is challenging^5,6^.

One approach to care delivery that has received greater focus during the pandemic is remote patient monitoring (RPM)^7–9^. Driving the uptake of this technology is the necessity to minimize in-person patient interactions as well as the need to leverage acute care facilities for the sickest patients and manage patients at home if possible^10^. The goal of RPM in the COVID-19 use-case is to keep healthcare professionals safe and optimize patient care^11–13^. For the most part, RPM in the context of COVID-19 has included intermittent monitoring (point measurements while the patient is awake) to assess oxygen saturation (pulse oximeter) and temperature (thermometer)^14,15^. Patients are then instructed to call their healthcare professional if the values for these measurements are outside the parameters established by the healthcare team. Interestingly, these clinical parameters, for both oxygen saturation and body temperature, have all demonstrated a likelihood of decompensation at a range of thresholds, making their clinical value equivocal^2,16–22^.

While commonly used and believed helpful for alerting physiologic decompensation^8^, intermittent active monitoring of SpO_2_ and body temperature are not without their challenges in a disease that manifests with a wide variety of signs and symptoms^23^. Pulse oximeters often do not provide accurate readings of oxygen saturations below 90% and have inconsistent repeated measurements^24,25^. Additionally, as SpO_2_ measurements rely on photoplethysmography technology, readings are sensitive to skin pigmentation and highly susceptible to motion artifact^26,27^. Therefore, people with darker skin tones may not be able to obtain accurate readings as well as patients with Parkinson’s or essential tremor. Though less sensitive to the irregularities of SpO_2_, fever has only been identified as a clinical symptom for less than a third of all hospitalizations for COVID-19^28,29^. Additionally, clinical definitions for fever and SpO_2_ are varied. Fever definitions range between prolonged elevation above 38 °C for at least 24 h^22^ to any temperature above 37.3 °C ^30^ or 39 °C^21^, while thresholds for low SpO_2_ levels could be as high as 95%^17^ to anything below 90%^20,31^. These varying definitions contribute to inconsistent findings when characterizing the case definition of COVID-19.

While both intermittent temperature and SpO_2_ monitoring may be incrementally beneficial, measuring other physiological features such as heart rate or respiration rate, particularly combined with patient ambulation, may provide greater insight into physiologic changes. Davis et al. found lead times for variations in physiological features following infection of non-human primates, finding significant heart rate increase approximately two days prior to a fever^32^. Other studies have found that respiratory rate changes may precede decreases in oxygen saturation^33–35^ and abnormalities of respiration have been identified as one the most important indications of clinical deterioration^36^.

In order to acquire and clinically manage a larger set of physiologic variables, like heart rate and respiratory rate, passive continuous remote patient monitoring (CRPM) could be advantageous to intermittent self-spot checking. Additionally, the continuous monitoring captures both ambulatory and resting physiological data. Numerous studies have identified the benefits of a steady data stream over spot-checking toward more timely alerting of patient degradation. Benefits include earlier interventions, better personnel allocation, shorter hospital stays, and decreased chance of hospital readmission^37–41^. Focusing on just intermittent monitoring of oxygen saturation and temperature rather than early changes in overall cardiorespiratory status through heart rate, respiratory rate, and other derived indices, it is likely that early warning signs of COVID-19 decompensation are being missed^42^.

Detection of early indicators of decompensation could be optimized through machine learning with continuous data streams. Numerous models have been trained and validated for predicting decompensation and diagnosis of COVID-19. Decompensation models have included predicting clinical outcomes, critical events, mortality, and hospitalization in the setting of COVID-19. However, each of these models relied on collection of laboratory panels, chest radiographs, patient reported symptoms, and/or computerized tomography scans^43–48^. To our knowledge, there have been no multivariate models developed using only non-invasive CRPM to predict decompensation due to COVID-19.

Therefore, we propose a model using only data collected continuously from a non-invasive wearable device, to create a COVID-19 decompensation index (CDI), which accurately predicts COVID-19 clinical deterioration, resulting in hospitalization. Continuous minute signals were windowed using a moving 24 h window, with a 1 h step size, timestamped to the end of the 24 h. The windowed signals were processed to create a feature vector on a once per hour cadence, using only the information in the past 24 h. Each hourly feature vector formed the input to the CDI model (see Fig. 6). To collect training data for the CDI, a clinical team leveraged CRPM as part of their standard of care, requiring participants to wear a biosensor to collect electrocardiogram and accelerometer data continuously for 28 days. If a clinical team member observed a participant’s physiology deteriorating (decompensation), participants were encouraged to return to seek care. Using this labeled data as the ground truth for decompensation, various features were engineered related to participants’ activity and cardiovascular system to predict a decompensation event. CDI, a gradient boosted model, was developed and outperformed current standard of care alerting systems of oxygen saturation and temperature monitoring. The study protocol from which this work was derived was the “Personalized Analytics and Wearable Biosensor Platform for Early Detection of COVID-19 Decompensation Study" or DeCODe: Detection of COVID-19 Decompensation)^49^.

## Results

### Participant Information

Four hundred COVID-19 positive participants were enrolled from University of Illinois Health System (UIH). Participants were adult patients (>18 years of age), and were recruited from two sources: (1) patients testing positive for COVID-19 in the outpatient setting and (2) patients who were admitted to the hospital with a diagnosis of COVID-19 and subsequently discharged to home convalescence. The cohort had a high percentage of individuals from traditionally underserved communities, including 46.2% Hispanic and 36.8% Non-Hispanic Black (see Table 1). The average age was 46 years old (±15.4), 67.2% female, with an average body mass index of 33.7 (±9.4). The most common co-morbidities were obesity (58.8%), hypertension (32.0%), and diabetes (26.2%). Participants were recruited from point of care testing centers, clinics, or the emergency department (360); however, 40 participants were enrolled post hospital discharge for COVID-19. Participants were expected to complete a total of 28 days of CRPM; however, averaged 22.2 ± 8.6 days of continuous data collection. Throughout monitoring, 25 participants were hospitalized due to complications of COVID-19. Eleven of the hospitalized participants reported to the emergency department (ED) independently while 14 were prompted to visit the ED by the study team. Treatments during hospitalization were varied, but primarily consisted of supplementary oxygen (15) and/or steroids (15). One participant required mechanical ventilation and one participant died after completing the study. After filtering for minimum data requirements, 308 participants were used to form the COVID-19 decompensation negative group, and 22 participants formed the COVID-19 decompensation positive group (“Methods”). Each COVID-19 decompensation event was scored using the WHO ordinal score^50^, where decompensation scores ranged from 3 (hospitalized, no oxygen therapy) to 8 (death).

**Table 1:** Demographics and co-mordibities of the participants in the DeCODe phase 1 study.

	Overall (400)	Negative (308)	Positive (22)
Age	46.0 (15.4)	45.2 (15.1)	55.3 (14.4)
BMI	33.7 (9.4)	33.6 (9.3)	35.6 (9.7)
Female	67.2%	65.3%	77.3%
Hispanic	46.2%	47.4%	50.0%
Non-Hispanic Black	36.8%	34.1%	36.4%
Non-Hispanic White	9.5%	9.7%	9.1%
Obese	58.8%	57.8%	72.7%
Diabetes	26.2%	26.9%	31.8%
Hypertension	32.0%	31.2%	50.0%
Serious heart condition	4.5%	3.6%	9.1%
COPD	1.8%	1.6%	4.5%
Smoking history	12.2%	12.0%	13.6%
Cancer history	3.2%	2.6%	4.5%
Moderate to severe asthma	16.5%	14.3%	27.3%

The negative and positive columns provide the information of participants used in the CDI K-fold validation. Age and BMI are listed as mean (standard deviation).

### Validation and modality comparisons

**Fig. 1:**
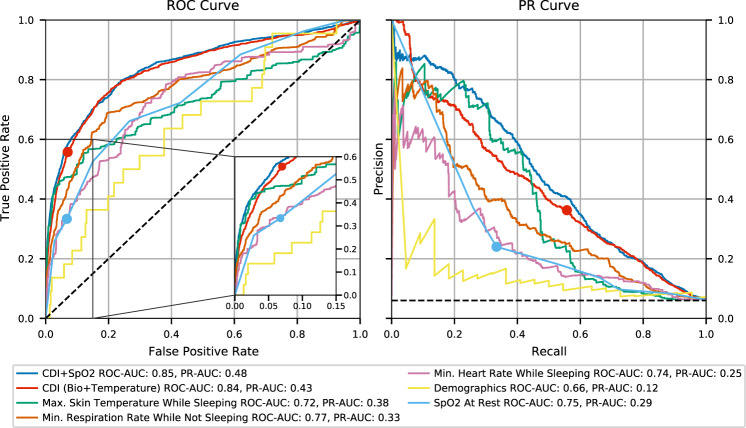
ROC and PR curves for selected models and univariate signals. The PR and ROC curves are weighted for equal participant contribution. The operating threshold for CDI and the 93% O_2_ threshold are marked as points on the curves.

The CDI model was trained using K-Fold cross validation. The overall area under the curve (AUC) of the receiver operating characteristic (ROC) of CDI was 0.84 (mean AUC across all folds was 0.85, 95% confidence interval (CI) 0.77–0.94), with an overall AUC of the precision-recall (PR) Curve of 0.43 (mean AUC across all folds was 0.48, 95% CI 0.33–0.62). All results were weighted for equal participant contribution to the ROC and PR curves. An operating threshold with a false positive rate of 7% was chosen to match the false positive rate of SpO_2_ at rest at a 93% O_2_ discrimination threshold. As seen in Fig. 1, the corresponding true positive rate at the 7% false positive rate is 55.7%, compared to 33.9% for SpO_2_ at rest. To understand CDI performance in the context of other types of data typically analyzed in a clinical setting, we compared CDI to several univariate features and alternative models. The best univariate features were determined by the largest ROC-AUC among the feature categories of temperature, respiration rate, and heart rate. Additional models of the CDI features with SpO_2_ and demographics were trained identically to the primary CDI model. Both CDI models produce the best overall ROC and PR curves, although the addition of SpO_2_ measurements into CDI improves the positive predictive value (PPV) of the model. We find the model built on demographic features of age, sex, race, height, weight, and body mass index to be less predictive of decompensation events than any model built on biosensor derived data. In general, the univariate features and model built from demographic data perform worse than the CDI models. However, maximum temperature while asleep does provide strong predictive value in the lower true positive rate (TPR) region of the ROC curve.

The performance of models built of various feature combinations are listed in Table 2. Only models utilizing features derived from the combination of biosensor derived features (ECG and acceleromety) achieve a ROC-AUC of greater than 0.8. Models utilizing features derived only from heart rate, respiration rate, actigraphy, and heart rate variability achieve ROC-AUC values from 0.69 to 0.71.

**Table 2:** Performance of univariate features and multivariate models.

	TPR	PPV	ROC-AUC
Bio + SpO_2_	57.0%	36.8%	0.85
CDI (Bio + Temperature) + SpO_2_	58.2%	37.2%	0.85
Bio + Feeling + SpO_2_	56.1%	36.4%	0.84
CDI (Bio + Temperature)	55.7%	36.4%	0.84
Bio + Feeling + SpO_2_ + Demographics	55.9%	36.4%	0.83
Bio + Feeling	48.4%	33.1%	0.82
Bio	50.9%	34.2%	0.82
Bio + Feeling + SpO_2_ + Temperature	44.9%	31.4%	0.81
Bio + Temperature + Demographics	44.9%	31.4%	0.81
Actigraphy only + Nighttime features	42.1%	30.1%	0.79
Min. respiration rate while not sleeping*	43.3%	30.6%	0.77
SpO_2_ at rest*	34.0%	23.8%	0.75
Min. heart rate while sleeping*	34.6%	25.6%	0.74
Max. Skin temperature while sleeping*	47.4%	31.5%	0.72
SpO_2_ after walking*	43.6%	31.0%	0.72
Nighttime features	22.7%	18.8%	0.72
HRV only	26.2%	21.2%	0.71
Actigraphy only	25.7%	20.8%	0.71
RR only	29.8%	23.3%	0.69
HR only	31.9%	24.6%	0.69
Feeling + SpO_2_	27.0%	21.6%	0.68
Temperature + SpO_2_	40.7%	29.3%	0.68
Demographics + Feeling + SpO_2_	14.7%	13.9%	0.67
Demographics	18.2%	15.4%	0.66

All statistics are weighted for equal participant contribution. TPR and PPV are given at 7% FPR threshold. All univariate features are marked with an asterisk (*). Bio refers to the top 50 features ultimately derived from ECG or Accelerometery signals, Feeling refers to the overall symptom survey response. Other feature groups represent models created using only the top 50 features from that particular source (i.e., the Actigraphy Only model uses only the top 50 actigraphy based features).

**Fig. 2:**
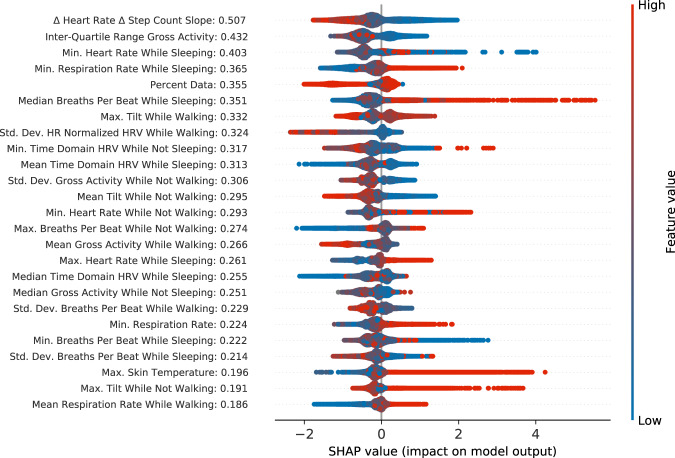
Shapley additive explanations (SHAP) scores of the most important 25 features, plotted as a beeswarm plot. Each point represents one datapoint in the training set, colored based on the relative value of the feature, where red is a high feature value, and blue is a low feature value. Points contributing to a positive (for hospitalization) decision have positive values, while those contributing to a negative (no hospitalization) decision have negative values. The mean absolute value of the SHAP scores is listed next to each feature name.

Tree based models like gradient boosted trees allow for more interpretable indications of what features were important for the model to reach its decisions. One way of measuring feature importance is Shapley additive explanations, or SHAP scores^51^. SHAP scores for the top 25 features are plotted in Fig. 2. We find low activity, high respiration rate, reduced heart rate variability, and high skin temperatures as important features for hospitalization prediction. Among the top 25 features, 20 were filtered statistical features, of which 9 were “while sleeping”, 2 were “while not sleeping”, 5 were “while walking”, and 5 were “while not walking”. We also find the interaction feature “the slope of delta heart rate to delta step count” as the most important individual feature. Intuitively, this feature can be understood as how dynamically one’s cardiorespiratory system can react to the physical demands of a given activity, where faster responses to the changes of physical demands may be indicative of better cardiorespiratory health. This feature has only mediocre discriminative value by itself, with a univariate ROC-AUC value of 0.62, compared to the second most important feature, the interquartile range of gross activity, which has a ROC-AUC of 0.77. We hypothesize that the slope of delta heart rate to delta step count is a feature indicative of overall cardiorespiratory function and helps the model contextualize the other features, thereby acting as a hospitalization risk factor, but individually is only slightly predictive of hospitalization.

While we did not have true external validation data in this phase 1 study, we perform two additional case study analyses using two datasets not used during training to test model performance. The first dataset was composed of previously collected data from participants who did not have COVID-19. The dataset was from 161 participants, representing almost 200,000 h of continuous biosensor data. This dataset was used to confirm the false positive rates of the K-Fold models, and that it matched the false positive rate of the overall CDI model. The false positive rate was set to 7% for the COVID-19 decompensation task, and the final model achieved a 7.4% FPR on the COVID-19 negative task, compared with a mean FPR of the 22-fold models of 6.8% (95% CI 5.8–7.8%).

On the second dataset, two participants who were hospitalized but were unable to be officially adjudicated and given a WHO score were analyzed as decompensation events with unknown WHO score (though likely ≥WHO 3). Using the overall trained CDI model, the overall true positive rate for both participants was an average of 87.5%, with a true positive rate during the last two days before hospitalization of 96.0%. Adding these two participants to the overall positive dataset, the overall TPR increases from 55.7 to 58.3%.

### Comparison to standard of care and remote patient monitoring

**Fig. 3:**
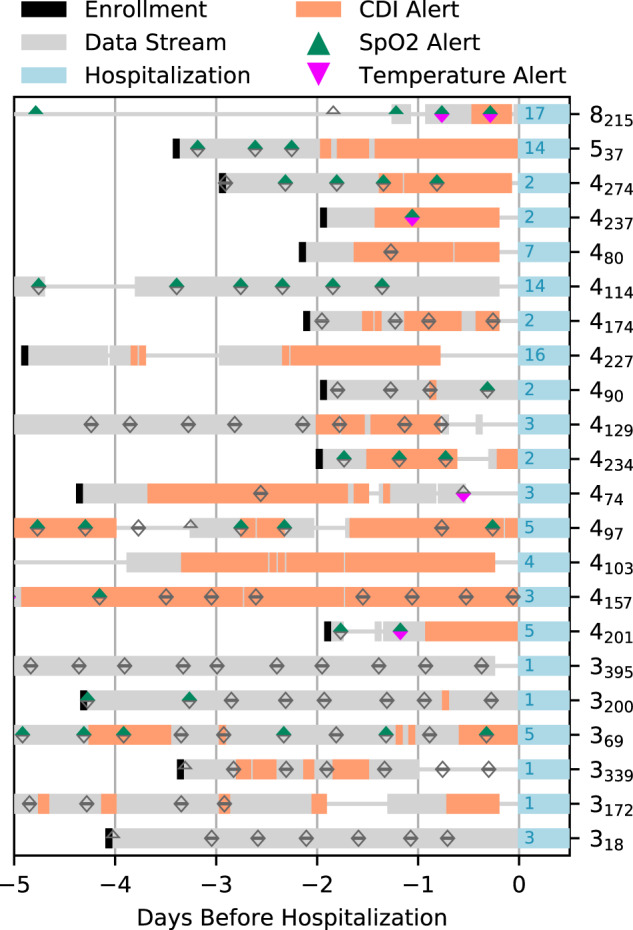
Comparison of simulated standard of care alerts to CDI alerts. Along the right edge is each participant’s WHO score and study ID (as a subscript), along with the number of days hospitalized in blue. Each SpO_2_ and temperature reading is shown as empty gray triangular indicators, each measurement above the alert indication (less than or equal to 93 O_2_, and greater than 36.11 °C) is colored.

The detection capabilities of CDI were compared to a simulated standard of care, where standard of care was defined as thresholded SpO_2_ and thresholded skin temperature values (oral temperature unavailable), as defined in the “Methods”. The comparison between CDI and simulated standard of care leading up to each analyzed hospitalization event is shown in Fig. 3. In the 2 days leading up to hospitalization, CDI alerts for 19 out of 22 participants, contrasted to 10 of 22 for simulated standard of care alerts. Four participants did not take any SpO_2_ measurements during the two days before hospitalization; all four participants had CDI alerts during the same time period. Only one participant had a standard of care alerts without also having a CDI alert in the last 2 days before hospitalization, and two participants had no alerts of either kind. As can also be seen in Fig. 3, CDI alerted far more frequently for participants with WHO scores of four or greater than those with WHO scores of 3. Participants with WHO scores of 3 had an average time in the hospital of 2 days, and those with WHO scores of 4 or higher had an average hospitalization time of 6.3 days. Temperature alerts were rarely triggered, despite the good predictive value of the daily maximum temperature, the temperature taken at an arbitrary patient-selected time point during the day seems to have low sensitivity.

**Fig. 4:**
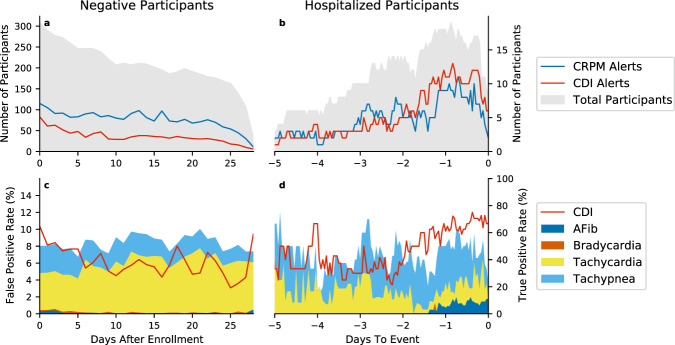
Comparison of a continuous remote monitoring system with clinical alerting rules to CDI. **a** The daily count of non-hospitalized participants with any false alerts. **b** The hourly count of alerts generated for hospitalized participants in the 5 days before hospitalization. In both **a**, *b* the light gray background denotes the number of participants with data at each timepoint. **c** The daily false positive rate of CDI and the component alerts. **d** The hourly true positive rate of CDI and the component alerts five days before hospitalization.

To further examine the potential benefits of using a machine learning model like CDI, we compared the detection capabilities of CDI to a CRPM system set to alert on conventional clinical rules. These CRPM alerts are non-specific to COVID-19, with every hour considered an alert if any of the underlying CRPM rules are met (“Methods”). The CRPM alerts compared to CDI based alerts can be seen in Fig. 4. Both CRPM and CDI alerts show relatively high true positive rates leading up to the events, although CDI does have a consistently higher TPR in the two days before hospitalization. The true positive rate of CDI gradually rises starting 2 days before the events, going from around a 40% TPR to a 70% TPR on the day of the event. However, the largest difference between CRPM and CDI alerts occur in the reduction in false positive rates among negative participants. In comparison to CDI, the simulated CRPM system alerted more frequently and across more individual participants.

## Discussion

While the majority of individuals infected by SARS-CoV-2 will ultimately not require hospitalization, up to 20% will^2^. The time from symptom onset to the need for hospitalization can extend beyond 10 days^52^, and the severity of an individual’s clinical course is rarely clear at the time of diagnosis. Accordingly, effective CRPM capabilities are critical to assuring patient safety while conserving scarce resources of both hospital beds and health care professionals. We show that through the use of a multi-parameter sensor patch in the outpatient setting that we were able to develop a machine learning based CDI model that has the potential to significantly improve the lead time and accuracy of identifying individuals requiring hospitalization due to progressive COVID-19, relative to what is routinely done in current remote monitoring programs.

The CDI model moves well beyond the current standard of care that depends on one or two population-based cut-offs (e.g., oxygen saturation <93%, or temperature >38 °C), captured only 2- to 3-times a day. Instead, using 17 source signals sampled every minute, which are further processed to create 361 features each hour, earlier signals of decompensation are detectable. While the continuous physiologic data available through a wireless patch sensor adds considerable value to intermittent spot checks, we have shown that the CDI model improved overall performance with a large reduction in false positive alerts relative to the sensor data alone. It is telling that the model features of greatest value were based on the interactions between individual features. For example, the relationship between heart rate during a given amount of activity may better track overall cardiorespiratory performance and characterize the impact of progressive COVID-19 on it.

The CDI model inherits the best attributes of the underlying features used to build the model. Features derived from respiration and temperature tend to show good discriminatory values in the low false positive region, as very high respiration rates and temperatures are quite predictive of decompensation events. Features derived from actigraphy signals are not able to predict decompensation events well, as those with COVID-19 already generally have low levels of activity, but those with high levels of activity can be ruled out for COVID-19 decompensation.

The CDI model shows an improvement over the standard of care patient initiated SpO_2_ and temperature checks (Fig. 3), particularly for participants with decompensation events of WHO ≥4, as 15 out of 16 participants had at least one CDI alert in the 2 days before their hospitalization. Four participants did not take any SpO_2_ measurements in the 2 days leading to their hospitalization, and eight participants did not take any SpO_2_ measurements during the 24 h before their hospitalization. This illustrates the importance of a monitoring system that does not require the participants to take action precisely at the time they are likely to feel the worst, but during which time their compliance is most critical. A CRPM system that passively collects biosensor data and can alert clinicians to potentially dangerous situations without the need for patient action could be an improvement over a system only utilizing SpO_2_ and temperature checks. Though, as seen in Fig. 4, simply using typical clinical rules based on remote monitoring data is likely to lead to alarm fatigue due to the high false positive rate. While simple thresholds on standard vital signs can provide decent COVID-19 decompensation detection, it is at the cost of a high false alert burden across many individual participants. Using a CDI model within a remote monitoring system could allow for the best of both worlds, achieving high detection capabilities, but with a suppressed false alarm rate over simpler clinical rules. However, while early detection of patient deterioration is an extremely important dimension of an effective CRPM approach, there are other dimensions that are just as important. For a CRPM platform to be adopted by clinicians it needs to be highly reliable, cost effective, easy to use, and enable clinicians to more efficiently manage a patient population. CRPM solutions that do not address all these dimensions are unlikely to become part of patient management clinical workflow. In addition, while we believe that digital health solutions have the potential to improve equity in healthcare, unless socioeconomic and educational barriers are proactively addressed, they could actually contribute to increasing inequity^53^.

Our work adds to a growing body of literature addressing remote monitoring in COVID-19. A recent systematic review identified 17 examples of published remote home monitoring models^54^. Virtually all incorporated some aspect of symptom tracking, most included some form of daily temperature checks, and 11 of the 17 included pulse oximetry. The methods of each model and outcomes tracked were too heterogeneous to allow for any meaningful conclusions regarding the efficacy and safety of any of these models but do help inform the scope of the problem. Looking at the totality of data from remote COVID-19 patient monitoring studies, the incidence rate of decompensation requiring at least an emergency department visit varied from as high as 36% to as low as 2.6%^55,56^. In one well-characterized study involving 2348 people enrolled in a twice-daily text messaging symptom tracker study, 23% required some escalation of care, with 8.6% returning to the emergency department and 42% of those individuals requiring hospitalization^57^. For COVID-19 positive individuals in whom deterioration is recognized earlier, proven therapies such as monoclonal antibody cocktails that have been shown to decrease hospitalization and death by 70%, can be initiated sooner in those most likely to benefit the greatest^58^. A CDI model used within a CRPM system may provide valuable lead time before hospitalization, leading to better patient outcomes.

The pandemic has also served to accelerate the utilization of digital health technologies in order to better care for individuals without the typical requirement of a visit to a brick-and-mortar healthcare facility. Smartphone apps, and SMS-based messaging programs allow people to enter their overall health and any symptoms on a daily basis that can be monitored and acted upon by healthcare professionals is one such method^57,59^. Symptom tracking alone is subjective and will be inconsistent between people, and would also miss individuals with “silent hypoxia,” a phenomenon described in COVID-19^60^. As noted earlier, most remote monitoring programs incorporate temperature checks, but a fever has turned out to not be the hallmark for COVID-19 infection-initially thought with some case series finding a fever at the time of initial hospitalization in less than one-third of patients, and only just over a quarter of COVID-19 positive nursing home residents^28,29^. Some programs have also incorporated home pulse oximetry, which has been found in one study of 77 COVID-19 outpatients to be helpful in prompting a return to the emergency department for an asymptomatic decrease in oxygen saturation and to reassure others that they did not need to return to the emergency department when their oxygen saturation was unchanged^29^. However, beyond the inherent limitations of intermittent self-testing with a pulse oximeter, there remain many practical issues around their reliability in large populations including their accuracy at lower oxygen levels, in settings of low pulsatile flow, and most especially racial bias leading to higher levels of unrecognized hypoxemia in Black relative to White populations^24,61^. Our work specifically sought to recruit from traditionally underserved communities of color to avoid such racial bias, with the study cohort consisting of 46.2% Hispanic and 36.8% Non-Hispanic Black participants.

This work has several limitations. Perhaps the largest limitation is the relatively small number of decompensation events. Because of the small number of decompensation events, we could not use a true holdout test set, and although we took several precautions against overfitting, given the small size of the positive dataset, we cannot entirely guard against it. However, this was only the phase I pilot part of the study, and we expect additional model validation to occur during the larger phase II part of the study. An additional limitation to the predictive performance of the CDI model is that we had no information about participants’ healthy, pre-COVID-19 baselines. For example, prior research has found significant inter-individual variability in “normal" resting heart rates^62^. With up to a 70-BPM difference between individuals’ average resting heart rates, any non-individualized heart rate-based features will lack the precision truly possible. Other limitations include: the temperature sensor used in the biosensor device was a skin temperature measurement and not a true oral temperature, and that the study cohort skewed mostly female.

We show a multivariate COVID-19 Decompensation Index capable of outperforming several standard clinical monitoring modalities using only data collected from wearable biosensors. CDI shows promise as a means of extending continuous remote patient monitoring capabilities, particularly above the use of intermittent, patient driven univariate monitoring. While cross-validation and hundreds of thousands of hours of data were used to minimize the likelihood of overfitting, a larger sample size is necessary to further validate the accuracy of the model. Future validation work is currently in progress as part of the phase 2 DeCODe study where CDI will be evaluated extensively to continue optimizing the use of CRPM^49^.

## Methods

### Data collection

Data were collected to develop and test a COVID-19 Decompensation Index from participants during the phase I portion of the Detection of COVID-19 Decompensation (DeCODe) study^63^. DeCODe was supported by the NIH National Cancer Institute (NCT04575532). The DeCODe study is a prospective, non-randomized, open-label study, with the primary outcome to develop a machine learning model to predict COVID-19 decompensation events, with secondary outcomes evaluating the feasibility of the pinpointIQ™ continuous remote patient monitoring system. Participants were adult patients (≥18 years of age) in the University of Illinois Health System. Patients were recruited from two sources: (1) patients testing positive for COVID-19 in the outpatient setting and (2) patients who were admitted to the hospital with a diagnosis of COVID-19 and subsequently discharged to home convalescence. The enrollment target was 400 participants for the phase I study. Participants were monitored for 28 days using the physIQ pinpointIQ™ platform to capture continuous biosensor data from the VitalConnect VitalPatch^®^ chest patch biosensor. The biosensor is an FDA cleared device that acquires 5–7 days of continuous raw 125 Hz ECG, 50 Hz triaxial accelerometer, and 0.25 Hz skin temperature data from which physiological features are derived by the pinpointIQ™ platform. These derived features drove the development of the CDI model.

Participants responded to symptom questionnaires and performed manual finger pulse oximetry measurements. The finger pulse oximeter sensor provided was the Proactive, Protekt^®^ Finger Pulse Oximeter 20110. Results of both were captured by the pinpointIQ™ smartphone app. Prompts to respond to the symptom questionnaire and to perform pulse oximeter measurements were pushed to the study smartphone twice daily. Each pulse oximetry measurement prompt included two measurements. The prompts for the symptom questionnaire and pulse oximetry measurements are shown below.

1. Symptom questionnaire:
  How are you feeling since the last time you completed the survey?
    OPTIONS: “Better”; “Worse”; “No Change”.
2. SpO_2_ measurements:
  Record your SpO_2_ at rest:
    Options: “100%”; “99%”; “98%”; “97%”; “96%”; “95%”; “94%”; “93%”; “92% or Below”.
  Record your SpO_2_ while walking:
    Options: “100%”; “99%”; “98%”; “97%”; “96%”; “95%”; “94%”; “93%”; “92% or Below”.

Once enrolled, participants were provided with a study kit containing a smartphone, finger pulse oximeter, a supply of biosensors, and instructions on the study procedures to follow and how to uses the devices.

### Exclusion criteria

Individuals were excluded with known allergic reactions to components of the biosensor hydrocolloid gel adhesives; or with cognitive or physical limitations that could have limited their ability to fully follow study procedures. Individuals that did not speak or read English or Spanish were also excluded.

### Ethical approval

This study was approved by the University of Illinois, Chicago Institutional Review Board. All participants provided written informed consent prior to enrolment in the study.

### COVID-19 decompensation event adjudication

A “clinical event” was defined as an escalation of care from home-based remote monitoring to a higher level of care. For example, if during monitoring the clinical user identifies that the patient is worsening and they need acute care, this is defined as a clinical event. Health record documentation was gathered from care facilities on any clinical event and was used for final adjudication as a COVID-19-related event. The adjudication consisted of review of the electronic health record of the participant experiencing the clinical event and two emergency department physicians independently deciding on whether the event was a “COVID-19 clinical event” or “non-COVID-19 clinical event”. If the two opinions did not agree, the case was reviewed by a third emergency department physician and final decision was made. Finally, a COVID-19 clinical event was defined to be a “Decompensation Event” if a hospitalized patient reached a maximum WHO Ordinal Scale for Clinical Improvement (WHO OSCI) score of 3 or more during their hospitalization. A WHO Score of 3 corresponds to “Hospitalization, no oxygen therapy”^50^. The COVID-19 Decompensation events served as the positive cases for the development of CDI.

### Ground truth formatting and analysis methods

**Fig. 5:**
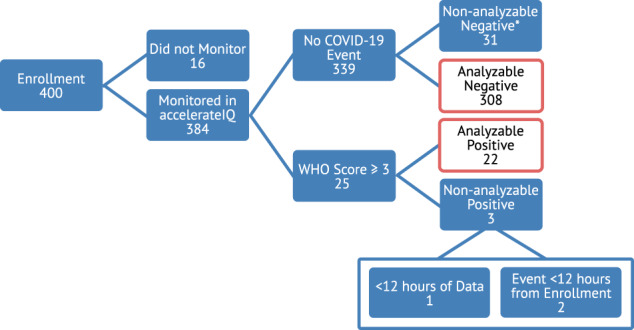
The analysis flowchart from 400 enrolled to 330 ultimately analyzed participants. The asterisk denotes 31 participants without COVID-19 events who did not have enough data to analyze, and were excluded from analysis.

Clinical outcomes were formatted for scoring of a classifier by the following steps. All participants who did not experience a COVID-19 event of any kind based on the WHO Ordinal Scale^50^ and who had at least 12 h of continuous biosensor data formed the negative group (308 participants). The entirety of the data collected from enrollment to completion or withdrawal from each event-negative participant was analyzed. All participants who experienced only a COVID-19 event(s) with a WHO score less than 3 (16 participants) were removed from analysis. WHO scores below 3 are not hospitalized and thus are not decompensation events, but conversely do not fit into the event negative group as many sought medical treatment, though they were not sick enough to be admitted to the hospital. Data from participants with WHO scores of 3 or above (25 participants) which had enough data for analysis (22 participants) were analyzed up until their first event with a WHO score of 3 or above. Two participants had more than one event with a WHO score of 3 or above; these additional events occurred two days after being discharged from the hospital from their first event and were not analyzed. We defined the positive time window for each event as up to two weeks before their hospitalization time, ending at the time of hospitalization. Any WHO scores less than 3 that occurred during the positive time window were ignored. The participant clinical flowchart can be seen in Fig. 5.

Each hour of data from participants without events and each hour of data from the positive time window of participants with events were treated as ground truth targets, with target values of 0 for the negative class, and values of 1 for the positive class. Any hour of ground truth target which did not have a corresponding CDI value (for example, during the first 12 h of monitoring) was not considered in the CDI performance analysis. To account for the variance in the amount of data collected by each participant and to ensure reported performance was not skewed by participants with more data than others, all confusion matrix calculations were weighted so that the contribution from each participant was equal. Weighting the confusion matrix in this manner results in a maximum true negative of 308, and a maximum true positive of 22.

### Feature extraction

**Fig. 6:**
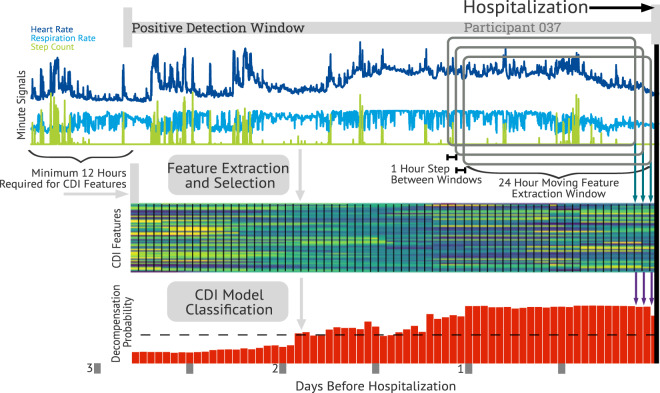
Processing steps from wearable features to CDI outputs. The source minute features (3 of 17 shown above) are windowed using a moving 24 h window with a 1 h step size. Each windowed set of minute features are processed to produce a feature vector, and timestamped to the end of the 24 h window. Each feature vector is independently passed to the CDI model to produce a decompensation probability at a cadence of once per hour. The positive detection window is defined as up to 14 days before the time of hospitalization and is evaluated at corresponding CDI decisions.

The VitalConnect VitalPatch^®^ device records raw ECG, 3-axis Accelerometer, and skin temperature, which is processed by the physIQ platform to produce 17 source signals at a once per minute sampling rate, listed in Table 3. The minute level signals were further processed to create a 361-length feature vector using a 24 h analysis windows with a 1 h slide. Feature extraction for each window would occur if at least 12 out of the maximum 24 h of data was available (that is, if at least 770-min samples were available in a given 24 h period). Provided the data requirements were met, the 17 source signals would produce 361 features at a hourly cadence, timestamped to the end of the 24 h window. Figure 6 shows the overall processing steps from minute signals to CDI probability. The feature extraction steps to produce the 361 features for the CDI model are defined below.

**Table 3:** Minute signals used as the source signals for feature extraction in the CDI model.

Signal name	Signal description
Heart rate	10% Trim mean average of beat to beat heart rate values
Time domain heart rate variability	10% Trim mean average of time domain heart rate variability
Respiration rate	10% Trim mean average of respiration rate
Count of magnitude	Average of activity count using the 3-axis accelerometer vector magnitude
Gross activity	Average of root mean squared of 3-axis accelerometer signal
Magnitude of uni counts	Average of vector magnitude of 3-axis univariate accelerometer activity counts
A-Fib percent	Percent of time classified as exhibiting atrial fibrillation or atrial flutter
Sleep	Percent of time classified as sleeping
Step count	Number of steps
Tilt	10% Trim mean of tilt angle of the torso (where 90 degrees is upright)
Trailing activity	Average of gross activity after application of a 3 minute moving average filter
Walk percent	Percent of time classified as ambulation (inclusive of walking and running)
Skin temperature	Median skin temperature, in degrees celcius
Activity residual	10% Trim mean average of trailing activity residual
Heart rate residual	10% Trim mean average of heart rate residual
Respiration rate residual	10% Trim mean average of respiration rate residual
MCI	10% TrIm Mean Average of MCI (multivariate change index)[?]

The minute source signals are generated by the pinpointIQ™ platform.

Two additional minute features, here named “Breaths Per Beat” and “HRV Normalized by HR,” were created by dividing minute respiration rate by minute heart rate, and by dividing the minute time domain heart rate variability by heart rate, respectively. A set of six statistical operations (median, mean, standard deviation, 1st percentile, 99th percentile, and interquartile range) were applied to the minute signals as listed in the Statistical Feature Group in Table 4. The 1st and 99th percentile were used in lieu of minimum and maximum to avoid any erroneous true minimum or maximum values. Statistical features were also calculated on a subset of signals while filtered for certain activities. The filtered statistical features were filtered such that the operations of median, mean, standard deviation, 1st percentile, and 99th percentile were calculated during periods of walking, not walking, sleeping, and not sleeping. Minutes were classified as walking or sleeping if more than 50% of the minute was classified as walking or sleeping.

**Table 4:** Feature extraction transforms from 17 source signals to 361 features.

Feature Group	Number of Features	Input Signals	Description
Statistical	102	Count of magnitude, gross activity, heart rate, time domain heart rate variability, magnitude of Uni counts, A-Fib percent, sleep, respiration rate, step count, tilt, trailing activity, walk percent, skin temperature, heart rate residual, activity residual, respiration rate residual, MCI	The following statistical operations applied to each of the input signals: median, mean, standard deviation, 99th percentile, 1st percentile, interquartile range
Filtered statistical	240	Gross activity, heart rate, time domain heart rate variability, respiration rate, step count*, tilt*, skin temperature, breaths per beat, HRV normalized by HR, heart rate residual, activity residual, respiration rate residual, MCI	Applies the statistical functions median, mean, standard deviation, 99th percentile, 1st percentile to each of the input signals when filtered by the following conditions: (1) while walking, (2) while not walking, (3) while sleeping, (4) while not sleeping. Signals marked with * indicate features only used in conditions 1 and 2.
Weighted average	4	Step count, heart rate, time domain heart rate variability, respiration rate, breaths per beat	Weighted average of input signal using the corresponding step count as the signal weight.
Interaction	8	Step count, heart rate, time domain heart rate variability, respiration rate, breaths per beat	Slope and intercept of linear regression fit to step count against the other input signals, with step count as the dependant variable. Minutes with step count values of zero are removed before fitting the linear regression.
Delta interaction	4	Step count, heart rate, time domain heart rate variability, respiration rate, breaths per beat	Slope of linear regression fit to first order difference of step counts against the first order difference of the other input signals. Minutes with 0 step count first order difference are removed before fitting the linear regression.
Sleep	2	Sleep	Number of awakenings (count of transitions between asleep and awake state), and number of awakenings per hour of sleep.
Data quality	1	ECG Signal quality index	Amount of high quality ECG data per window
Total features	361		

Each feature group is listed along with the input signals and a description of transformations to yield the resultant features. All features are calculated over a 24 h time window with a 1 h step between each window, yielding a 361-length feature vector every hour.

Inspired by Mishra, et al.^64^, who found that the ratio of heart rate and step count was a predictive feature of COVID-19 infection, we developed a set of features to capture interactions between minute step counts and vital signs. The first set of features, called the Weighted Average Features, in Table 4 are features found by taking the weighted average of input signals with step counts as the weighting variable, according to the following weighting formula in Equation (1).

1 $${{weightedSignal}}=\frac{\sum {{Signal}}\cdot {{stepCount}}}{\sum {{stepCount}}}$$

The second set of features, called the interaction and delta interaction features in Table 4 are features found by calculating the slope and intercept of a linear regression of step count against heart rate, time domain heart rate variability (HRV), respiration rate, and breaths per beat, with step count as the independent variable. Step counts and the corresponding signal values when step counts were zero were not used to calculate the linear regression parameters. Additionally, the same linear regression procedure was repeated to calculate the linear regression slope on the first order differences of step count and the first order difference of the other signals.

Sleep quality and sleep apnea have been found to be correlated with COVID-19 outcomes^64,65^; to capture some sleep disturbance information, we calculated two sleep disturbance features in the CDI model. These were calculated by determining the number of awakenings, computed by counting the number of transitions from sleep to awake state, and the number of awakenings per hour of sleep, where a given minute was classified as sleeping if more than 50% of the minute was the sleep state.

One data quality feature was calculated by summing over an ECG signal quality index to calculate the percent of good quality data with respect to the total possible amount of data per day.

Other features not derived from biosensor data like demographics, survey responses, and SpO_2_ were interpolated with zero-order interpolation and timestamped to the end of each hour. Survey responses and SpO_2_ responses were interpolated for up to 24 h past the time of their original measurement.

### Model training

In this work, we chose to use gradient boosted decision trees^66^ as our classifier, as gradient boosted trees achieve high levels of performance and can provide greater insight into their decisions than other black box machine learning models, which is a desired attribute in the medical field. The principal difficulty in modeling COVID-19 decompensation was the large class imbalance, and the relative rarity of pre-hospitalization data. Of the 330 participants ultimately analyzed in this study, only 22 of them had analyzable events. The median amount of time from enrollment to COVID-19 decompensation event was 4 days, which was far smaller than the median number of days (26 days) of data collected by each negative participant. As a result, the total positive data was 1.4% of the total data. Given the small amount of positive data available, we were concerned with the possibility of overfitting on the dataset and took several steps to safeguard against overfitting and check that it did not occur.

We separated our modeling steps into (1) feature and hyperparameter selection, (2) K-Fold training of the CDI model, and (3) validation of K-Fold trained models on additional datasets. To help avoid overfitting on the decompensation detection task, we performed hyperparameter selection using a related but easier task of differentiating participants with a COVID-19 diagnosis from those without a COVID-19 diagnosis, using a previously collected dataset of roughly equal size to the DeCODe study, with the hypothesis that similar features and hyperparameters for the COVID-19 vs non-COVID-19 task would transfer to the task of COVID-19 decompensation detection. After selection of the hyperparameters, we used K-Fold model training and validation to produce performance estimates for decompensation classification, then trained a single model on all the training data. Finally, to validate the model we turned back to the non-COVID-19 data used in step 1, and though that data only contained examples negative for COVID-19 decompensation, it allows for confirmation that the false positive rates match the expected false positive rates found in the K-Fold model validation procedure.

To reduce the likelihood of overfitting on the COVID-19 decompensation detection task, we performed feature selection and XGBoost hyperparameter selection on the related task of classifying COVID-19 positive vs COVID-19 negative participants. The COVID-19 positive dataset was the entirety of the DeCODe Phase 1 dataset, and the COVID-19 negative dataset was a dataset of previously collected data from 161 participants, consisting of almost 200,000 h of continuous biosensor data. Feature selection was performed by training an XGBoost model and ranking the features by SHAP importance^51^. We selected the top 50 features based on SHAP importance and selected the rest of the model hyperparameters using a 5-fold grid search, based on the number of participants.

We applied a modified K-Fold validation approach, with K equal to the number of positive participants (22), such that each fold had one positive participant, and 1/22nd of the negative participants. On each iteration, 21 out of the 22 folds were used to train the model, with the remaining fold used for validation. For each training iteration, 2 days of data before events from participants with only WHO scores of two were added to the positive training data to help provide additional data. All features were z-scored based on the mean and standard deviation parameters calculated for each training iteration. To help combat class imbalance, samples during training were weighted with increasing importance leading up to the date of the COVID-19 decompensation. This weighting was done based on the hypothesis that in each positive window, the certainty that the COVID-19 event is happening grows as the time of actual event grows closer. Conversely, negative participants’ data were weighted with importance as decreasing from the date of enrollment, as we wanted the model to focus more on time periods where their disease severity is likely worse than toward the end of their data collection, when they likely would have largely recovered from the disease. The final model was trained using all 22 folds.

In an effort to further guard against overfitting, we performed an additional validation of the false positive rate of the K-Fold models and the final model trained on all the training data on the COVID-19 negative dataset used to select the model hyperparameters. Using this procedure allows verification that firstly the false positive rate of the final model is consistent with the false positive rates produced by each of the 22 models trained in the K-Fold validation, and secondly that the false positive rate on the COVID-19 negative dataset is consistent with the false positive rate achieved on the COVID-19 decompensation task.

Furthermore, we used two hospitalized participants data as an additional small positive dataset. These two participants could not be adjudicated and given a WHO score, as their medical records were inaccessible to the UIH medical system, but were likely hospitalized as a result of COVID-19.

### Experiments

We conducted several additional experiments to compare the CDI model to available standard and care and remote patient monitoring without using machine learning.

Comparisons to the CDI model were done in one of two ways, either a comparison to a univariate feature analysis, or in a multivariate feature analysis by building a new XGBoost model. The univariate feature comparison was created by using the feature of interest directly in the ROC analysis.

The multivariate feature analyses follow the same modeling steps as described above in “Model training”. However, instead of using the 50 best features without restriction, the model would use the 50 best with certain types of features removed. For example, a model only using actigraphy features would select the top 50 actigraphy features from the full list of ranked features used for CDI. The same hyperparameters used for the CDI model were used in all other multivariate model comparisons.

Simulated standard of care was defined as patient initiated SpO_2_ and temperature readings, which would alert if either are above typical clinical thresholds. An SpO_2_ alert was considered triggered if the measured SpO_2_ value at rest was less than or equal to 93%. Patient initiated temperature alerts were simulated by thresholding the skin temperature value occurring at the same time as the SpO_2_ measurement. Since skin temperature is lower overall than oral or core body temperature, standard clinical oral temperature thresholds were not valid thresholds. Instead, we considered a skin temperature reading to be an alert if the value was greater 36.11 °C, which is the 90th percentile of the temperature readings in the study.

We wanted to compare CDI to continuous remote patient monitoring to better understand the differences between a machine learning model derived from the same information, compared to a set of clinical rules. The CRPM rules were computed each hour, and an alert was considered triggered if any of the following conditions were met:

- Tachypnea rule: alert if the average respiration rate while not walking is greater than 26 breaths per minute.
- Tachycardia rule: alert if heart rate is greater than 100 beats per minute while not walking for more than 40 min per hour
- Atrial fibrillation rule: alert if AFib is detected for more than 40 min per hour.
- Bradycardia rule: alert if heart rate is lower than 45 beats per minute for more than 48 min per hour or if heart rate is lower than 52 beats per minute for more than 48 min per hour while awake.

### Reporting summary

Further information on research design is available in the Nature Research Reporting Summary linked to this article.

## Supplementary information

Reporting summary

## Data Availability

The data that support the findings of this study are available from the Digital Health Technologies Data Hub as well as the NIH RADx data hub (https://rapier.ll.mit.edu/studies). Users will be required to electronically sign a data use agreement. Each dataset will have its own DOI. Data will be available for general research use. Aggregate data analyzed in this study are available from the corresponding author on reasonable request.

## Code availability

The code used to train and validate the model, as well as perform the analyses, are available at the Digital Health Technologies Data Hub. They are also available from the corresponding author on reasonable request. All analyses were performed using Python 3.8, with xgboost==1.2.1, and shap==0.37.0.
