# Supplementary material for: Wearable sensor derived decompensation index for continuous remote monitoring of COVID-19 diagnosed patients
Source: NPJ Digit Med. 2021 Nov 8;4:155. doi: 10.1038/s41746-021-00527-z (PMC8576003; doi:10.1038/s41746-021-00527-z)
Supplement: Supplementary file 1 — Reporting summary [file 41746_2021_527_MOESM1_ESM.pdf]

nature portfolio

Corresponding author(s): Stephan Wegerich

Last updated by author(s): Jul 28, 2021

Reporting Summary

Nature Portfolio wishes to improve the reproducibility of the work that we publish. This form provides structure for consistency and transparency in reporting. For further information on Nature Portfolio policies, see our Editorial Policies and the Editorial Policy Checklist.

Statistics

For all statistical analyses, confirm that the following items are present in the figure legend, table legend, main text, or Methods section.

n/a	Confirmed	
☐	☒	The exact sample size (*n*) for each experimental group/condition, given as a discrete number and unit of measurement
☐	☒	A statement on whether measurements were taken from distinct samples or whether the same sample was measured repeatedly
☒	☐	The statistical test(s) used AND whether they are one- or two-sided<br>*Only common tests should be described solely by name; describe more complex techniques in the Methods section.*
☐	☒	A description of all covariates tested
☒	☐	A description of any assumptions or corrections, such as tests of normality and adjustment for multiple comparisons
☐	☒	A full description of the statistical parameters including central tendency (e.g. means) or other basic estimates (e.g. regression coefficient) AND variation (e.g. standard deviation) or associated estimates of uncertainty (e.g. confidence intervals)
☒	☐	For null hypothesis testing, the test statistic (e.g. *F*, *t*, *r*) with confidence intervals, effect sizes, degrees of freedom and *P* value noted<br>*Give P values as exact values whenever suitable.*
☒	☐	For Bayesian analysis, information on the choice of priors and Markov chain Monte Carlo settings
☒	☐	For hierarchical and complex designs, identification of the appropriate level for tests and full reporting of outcomes
☒	☐	Estimates of effect sizes (e.g. Cohen's *d*, Pearson's *r*), indicating how they were calculated

Our web collection on statistics for biologists contains articles on many of the points above.

Software and code

Policy information about availability of computer code

Data collection	Data was collected using the physIQ pinpointIQ v4.6.11 commercial software.
Data analysis	Data analysis were performed using python 3.8.8, with the following open source libraries:<br>matplotlib==3.3.3,pandas==1.1.4,seaborn==0.11.1,xgboost==1.2.1,shap==0.37.0,scikit-learn==0.22.0,numpy==1.18.0,scipy==1.4.1

For manuscripts utilizing custom algorithms or software that are central to the research but not yet described in published literature, software must be made available to editors and reviewers. We strongly encourage code deposition in a community repository (e.g. GitHub). See the Nature Portfolio guidelines for submitting code & software for further information.

Data

Policy information about availability of data

All manuscripts must include a data availability statement. This statement should provide the following information, where applicable:

- Accession codes, unique identifiers, or web links for publicly available datasets
- A description of any restrictions on data availability
- For clinical datasets or third party data, please ensure that the statement adheres to our policy

The data that support the findings of this study are available from the Digital Health Technologies Data Hub as well as the NIH RADx data hub. Users will be required to electronically sign a data use agreement. Each dataset will have its own DOI. Data will be available for general research use.

Field-specific reporting

Please select the one below that is the best fit for your research. If you are not sure, read the appropriate sections before making your selection.

☒ Life sciences ☐ Behavioural & social sciences ☐ Ecological, evolutionary & environmental sciences

For a reference copy of the document with all sections, see nature.com/documents/nr-reporting-summary-flat.pdf

Life sciences study design

All studies must disclose on these points even when the disclosure is negative.

Sample size	We assumed an event rate of 7.5% based on recent readmission rates for COVID-19 patients. Our target CDI performance was AUC = 0.75 with significance a = 0.05 and power 1 – ß = 0.80. The sample size required to achieve this performance is 12 positive cases, and 148 negative cases for a total sample size of 160. Our proposed sample size of 400 during Phase I enabled us to partition the data into development (for training) and holdout (for performance assessment) subsets.
Data exclusions	Data from 50 participants were excluded due to insufficient data to analyze. The data analysis required the participant to have at least 12 hours of data, which was not met by those participants (16 had no data, 34 had less than 12 hours). The exclusion criteria was not pre-established as the minimum amount of time required depends on the specific machine learning method used, and could not have been precisely defined before the study.
Replication	Reproducibility was confirmed through k-fold cross validation, in addition to confirming expected classification performance on additional "case study" datasets not used during the k-fold validation procedure.
Randomization	Randomization was not applicable in this study. All subjects were COVID positive subjects.
Blinding	No blinding was necessary in this study because the performance assessment approach defined for the study was based on k-fold bootstrapping methods which requires a-priori knowledge of the class each subject belongs to.

Reporting for specific materials, systems and methods

We require information from authors about some types of materials, experimental systems and methods used in many studies. Here, indicate whether each material, system or method listed is relevant to your study. If you are not sure if a list item applies to your research, read the appropriate section before selecting a response.

Materials & experimental systems

n/a	Involved in the study	
☒	☐	Antibodies
☒	☐	Eukaryotic cell lines
☒	☐	Palaeontology and archaeology
☒	☐	Animals and other organisms
☐	☒	Human research participants
☐	☒	Clinical data
☒	☐	Dual use research of concern

Methods

n/a	Involved in the study	
☒	☐	ChIP-seq
☒	☐	Flow cytometry
☒	☐	MRI-based neuroimaging

Human research participants

Policy information about studies involving human research participants

Population characteristics	Participants were adult patients (>= 18 years of age) in the University of Illinois Health System. Patients were recruited from two sources: 1) patients testing positive for COVID-19 in the outpatient setting and 2) patients who were admitted to the hospital with a diagnosis of COVID-19 and subsequently discharged to home convalescence.
Recruitment	Participants were recruited from point of care testing sites, the emergency department and hospitalized patients who were COVID-19 positive. The research team had access to testing results and could approach the patient for possible participation. Bias could be introduced because those that agreed to participate may have been more motivated to participate in their own health care by being monitored in the program (they sought testing or care from the institution). Bias may have also been introduced as symptom-free individuals may not have sought testing at all, therefore, healthier participants may not have been included in the sample.
Ethics oversight	This study was approved by the University of Illinois, Chicago Institutional Review Board

Note that full information on the approval of the study protocol must also be provided in the manuscript.

Clinical data

Policy information about clinical studies

All manuscripts should comply with the ICMJE guidelines for publication of clinical research and a completed CONSORT checklist must be included with all submissions.

Clinical trial registration	NCT04575532
Study protocol	A summary of the protocol is available on clinicaltrials.gov and description of the protocol can be found in the publication: Larimer K, Wegerich S, Splan J, Chestek D, Prendergast H, Vanden Hoek T<br>Personalized Analytics and a Wearable Biosensor Platform for Early Detection of COVID-19 Decompensation (DeCODe): Protocol for the Development of the COVID-19 Decompensation Index<br>JMIR Res Protoc 2021;10(5):e27271<br>The full protocol is not publicly available as it includes intellectual property.
Data collection	Data collection occurred at a large health system's point of care COVID-19 testing sites, in the emergency department, in affiliated clinics and in the hospital. Because most data was collected through the monitoring system, most data was collected remotely through the data analytics platform. First participant enrolled 10.5.20, began data collection 10.7.20. Last participant completed and data locked 12. 22.20
Outcomes	The primary outcome was a COVID-19 Decompensation Event which was defined to be a hospitalization due to COVID where a patient reached a maximum WHO Ordinal Scale for Clinical Improvement score of 3 or greater while hospitalized.
